# Cloning and Bioinformatics Analysis of *GhArfGAP* in Cotton (*Gossypium hirsutum*) Boll Abscission Layer With Ethylene Treatment

**DOI:** 10.3389/fpls.2022.841161

**Published:** 2022-06-24

**Authors:** Long Chen, AnFeng Liu, ZiWen Guo, Hui Jiang, Ling Luo, JunShan Gao, DaHui Li, SiHong Ye, Ning Guo

**Affiliations:** ^1^School of Life Sciences, Anhui Agricultural University, Hefei, China; ^2^Cotton Research Institute of Anhui Academy of Agricultural Sciences, Hefei, China

**Keywords:** upland cotton, abscission zone, *GhArfGAP*, bioinformatics, Golgi apparatus

## Abstract

With the continuous growth of the human population, the demand for fiber is also rising sharply. As one of the main fiber plants available globally, cotton fiber yield (*Gossypium hirsutum*) is affected by boll abscission, which is related to the formation of the abscission layer. Therefore, we explored the formation of the abscission layer in cotton. The formation of the abscission layer in the cotton boll stalk was promoted by exogenous ethylene. It was found that both the number of the Golgi apparatus and the number of stacking layers increased in the dissociated cells. The *GhArfGAP* gene family in cotton was screened by the bioinformatics method, and the species and evolutionary relationship of *the GhArfGAP* gene family were analyzed. qRT-PCR showed that *GhArfGAP13*, *GhArfGAP15*, *GhArfGAP25*, and *GhArfGAP34* in cotton had spatiotemporal-specific expression patterns. Subcellular localization suggested that *GhArfGAP25* played a role in the Golgi apparatus. The expression of *GhArfGAP25* in transgenic *Arabidopsis thaliana* is increased in the roots, stems, and leaves. Finally, we found that ethylene could induce the formation of the abscission layer in cotton. *GhArfGAP13, GhArfGAP15, GhArfGAP25*, and *GhArfGAP34* might regulate the changes in the Golgi apparatus in the abscission layer. Taken together, the findings provide new ideas for the study of the formation of cotton abscission.

## Introduction

Cotton (*Gossypium hirsutum*) is a major plant variety available in China and worldwide. It occupies an important place in the national economy. The main product of cotton is cotton fiber, an important raw material of the textile industry. Natural cotton fiber has many excellent characteristics that chemical cotton fiber does not have, which are as follows: good heat preservation, no pollution, strong moisture absorption, easy dyeing, and beautiful and comfortable products. Cottonseed is a by-product that can produce oil. Therefore, the demand for cotton is increasing year by year.

However, in the actual production of cotton, there are many factors affecting cotton yield; the shedding of bolls is an important factor that seriously restricts cotton yield. According to relevant reports, the shedding rate of cotton is generally 60∼70% (Mao et al., 2013), and in Northern Xinjiang, it is more than 80% (Yu and Wang, 1999). The shedding of bolls is related to the formation of the abscission layer. The formation of the abscission layer is a normal physiological phenomenon in plants, but this process is also complex, and related genes and exogenous hormones regulate it.

In tomatoes, the reported genes related to the formation of abscission are *jointless* (Roldan et al., 2017), *J2* (Yang et al., 2005), and *LS* (Schumacher et al., 1999). If these genes are mutated, the normal formation process of abscission will be destroyed, so the tomato pedicel cannot fall off or there are no petals in the flower. In rice, some genes have been confirmed to be involved in regulating the formation of abscission. *OsCPL1* (Ji et al., 2010) encodes the phosphatase region of CTD, which may limit the differentiation of the abscission layer by reducing the phosphatase activity, resulting in the weakening of rice, that is, seed shattering. However, *qSH1* (Konishi et al., 2006) promotes the formation of the basal abscission layer of the glume, and the seeds are easy to fall. Interestingly, *SH4* (Li and Olsen, 2016), similar to *qSH1*, is mutated so that the abscission layer cannot be formed, and the shattering phenotype is lost. In corn, *ZmSh1, Sh1-1*, and *Sh1-5.1* (Lin et al., 2012) are also involved in regulating the formation of the abscission layer. *GhBOP1* (Chang et al., 2015) is dominantly expressed in cotton abscission, and it may participate in the leaf abscission process by determining the differentiation of the abscission layer. In *Arabidopsis thaliana*, the *BOP1* (Hepworth et al., 2005) and *BOP2* (McKim et al., 2008) play an important role in the development of flower organ abscission, mitogen-activated protein kinase (MAPK) cascades play crucial roles in regulating abscission, and two MEKs (Cho et al., 2008), namely, MKK4 and MKK5, and their RNA interference transgenic lines show abscission deficiency.

In addition, exogenous hormones such as ethylene also affect the formation of abscission. Studies have shown that there is no obvious relationship between plant leaf abscission and endogenous ethylene content.

However, spraying ethylene can significantly promote plant leaf abscission (Abeles et al., 1992). The increase in ethylene content in cells induces the expression of related abscission genes to promote the increase in plant organ-related degradation enzymes, causes cell wall degradation, and leads to plant organ abscission. The related abscission genes include ethylene response gene PR (pathway-related proteins), the signal regulatory genes of plant organ abscission, and related genes of ethylene-induced plant organ abscission (Gao et al., 2013). Among them, the PR expression gene can be upregulated by ethylene. After mutations in ETR1 and EIN2 (Schaller, 2012) in the signal regulatory genes of plant organ abscission, insensitivity leads to delayed abscission. In addition, it is directly related to the abscission of plant organs. The related cellulose (Lashbrook et al., 1994; Bennett and Breakstrength, 1996; Ouyang et al., 2007) and polygalacturonase (Ogawa et al., 2009; Peng et al., 2013) genes can be directly affected by ethylene regulation.

Small GTP-binding protein family is a kind of GTP-binding protein family that generally exists in the eukaryotic cells. According to its protein structure and function, it is divided into five subfamilies: Ras, rho, RAB, ARF/SAR, and ran (Lin et al., 1996; Li et al., 1999). Small G proteins involved in physiological activities exist in two forms: The activated state bound to GTP and the non-activated state bound to GDP. When stimulated by the upstream signals, the GDP dissociates from the small G protein in a non-activated state, and the GTP binds to the small G protein in an activated state. Arf is a kind of small G protein. The transformation of Arf between the activated and non-activated states requires the regulation of at least two accessory proteins: the ArfGEF, which induces GDP to dissociate from small G protein to bind GTP (guanine nucleotide exchange factors, GEFs), with the small G protein in the activated state, and the ArfGAP (ADP-ribosylation factor GTPase-activating protein) of the small G protein that hydrolyzes the GTP into GDP for the small G protein (Bourne et al., 1990). Arf is involved in endocytic vesicle trafficking in plants. Vesicle transport is crucial for generating asymmetric cell division, which is the core of cell differentiation and multicellular development. It has been proven that Arf gene deletion and mutation will make *A. thaliana* show a non-developmental phenotype and affect the root phenotype (Xu and Scheres, 2005). The regulation of ARF on vesicle transport will directly affect the transmission of proteins to intracellular and extracellular compartments, the transmission of cellulose synthase to the plasma membrane, and the proximity of non-cellulose polymers to the cell wall, which will eventually affect cell division and expansion (Gebbie et al., 2005). ARF regulatory proteins also play an important role in regulating plant development. *A. thaliana* has an ArfGAP gene mutant, and it shows isotropically expanded, short, and branched root hairs with slow pollen tube elongation (Song et al., 2006). Some studies have shown that ARF and its regulatory proteins participate in the polar transport of auxin and control plant development (Steinmann et al., 1999; Sieburth et al., 2006). It can be seen from the above studies that ArfGAP, as a key protein regulating ARF activity, plays an important role in regulating biological growth and development. Unfortunately, there are only few studies on ArfGAP and the relationship between ArfGAP and the formation of the abscission layer in cotton with ethylene treatment. Hence, we performed this study to lay a foundation for reducing the boll abscission rate of cotton in advance.

## Materials and Methods

### Plant Materials

Ekangmian No. 9 (Jing55173), formally identified by Hubei Jingzhou Academy of Agricultural Sciences, was selected as an experimental variety. Seedlings were acquired from the Institute of Cotton Research of CAAS were grown in the National High-tech Agricultural Park of Anhui Agricultural University, Hefei, China. Cotton plants with consistent growth and good condition were selected as the experimental materials. These samples can be collected without permission.

### Bioinformatics Analysis of *GhArfGAP* Gene Family and GhArfGAP25 Protein

The TblastN (Wu et al., 2014) sequence was aligned between the amino acid sequence of the ArfGAP characteristic domain and the whole-genome sequence database of cotton, and the protein sequence of the candidate gene of *GhArfGAP* was screened. Then, Pfam and SMART programs (Ivica et al., 2008) were used to test whether it contains the GhArfGAP domain. The *GhArfGAP* gene sequence was aligned using the ClustalW tool (Thompson et al., 1994) of MEGA6.0 software to construct the phylogenetic tree. The exons and introns of *the GhArfGAP* gene family in cotton were analyzed by GSDs (Guo et al., 2007). The MEME online analysis tool (Bailey et al., 2015) was used to analyze the conserved motifs of GhArfGAP protein in cotton. MapInspect software was used to generate the distribution of all the *GhArfGAP* genes on chromosomes. TMHMM, SignalP4.1 Server, NPS, and SWISS-MODEL were used to analyze the structure of the GhArfGAP25 protein.

### Screening of Ethylene Concentration and Obtaining of the Boll Stalk Abscission Layer

This experimental method, referred to MA Nagao (Nagao and Sakai, 1988), was adjusted appropriately. Ethylene concentrations of 200 mg⋅L^–1^, 400 mg⋅L^–1^, and 800 mg⋅L^–1^ were applied on the cotton petiole abscission area. The control group was treated with water for 24 h, and the formation of the abscission layer was observed in the field to determine the optimum concentration used in the experiment. At 8:00 on the day of flowering, immediately after treatment with ethylene, the detached area of the flower stalk was cut with a clean and sharp blade, which was recorded as ET_0h_. In another flower, after treatment with water, the separate area of the flower stalk was cut as control, which was recorded as CK_0h_. In the same way, the rest were divided into the treatment group (ET) and the control group (CK), after treatment with ethylene and water, respectively, and samples were taken every 4 h. The materials were fixed with FAA, glutaraldehyde, and liquid nitrogen according to the different follow-up experiments and then stored in a –80°C refrigerator.

### Quantitative Real-Time and Semi-Quantitative RT-PCR

RNA was extracted from ET and CK using RNAprep Pure Plant Kit (TIANGEN, Beijing, China). Total RNA was reversed to cDNA using a FastQuant RT Kit (TIANGEN, Beijing, China). qRT-PCR was carried out on an Applied Biosystems 7500 Fast Real-Time PCR System (Life Technologies, Carlsbad, CA, United States) in a 20 μl volume containing 2 μl of cDNA, 0.8 μl of each primer, 10 μl of SYBR^®^Premix Ex Taq™ II(2×), and 6.4 μl of ddH_2_O. The PCR conditions were as follows: primary denaturation at 50°C for 2 min followed by 40 amplification cycles of 30 s at 95°C, 5 s at 95°C, and 20 s at 60°C. After the last cycle, the reaction was maintained at 72°C for 10 min. The melting curve analysis was performed to ensure there was no primer dimer formation. Information of the qRT-PCR primers for the gene expression analysis is listed in Supplementary Table 1. Three replicate assays were performed with independently isolated RNAs, and each RT-PCR was loaded in triplicate. The relative gene expression levels were calculated using the 2^–ΔΔCt^ method (Livaka and Schmittgen, 2002). Statistical analysis of the number of rosette leaves was performed using one-way ANOVA by the SPSS18 (Bryman and Cramer, 2012). Statistically significant differences (*P* < 0.05) are indicated by different lowercase letters and are used for quantitative fluorescence PCR. *UBQ7* (GenBank accession number: DQ116441) was used as an internal reference gene.

### Construction of Expression Vector

*GhArfGAP25* gene was ligated into pMD-18T plasmid to form pMD-18T-GhArfGAP25 plasmid, and then, pCambia1304 plasmid and pMD-18T-GhArfGAP25 plasmid were ligated to form pCambia1304-GhArfGAP25 recombinant plasmid. Later, the recombinant plasmid pCambia1304-GhArfGAP25 and the empty vector pCambia1304 were transformed into competent cells of the *Agrobacterium* strain, EHA105, by the electroporation method.

### Subcellular Localization

The *Agrobacterium* strain that contained the recombinant plasmid and the empty vector was coated on a double resistance LB solid medium with rifampicin and kanamycin to an OD_600_ value of the bacterial solution of about 1.5. It was centrifuged at 4,000 rpm and separated from the center after 10 min to collect the bacteria. Then, the bacteria were resuspended with the infection solution to make the OD_600_ value of the bacterial solution of about 0.8–1.0, and the infection solution was stored in a 4°C refrigerator for 3 h. Subsequently, the infection liquid was injected into tobacco leaves (*Nicotiana benthamiana*) with a sterile syringe, and dark culture was carried out in a light incubator. After 3 days, the infected tobacco leaves were placed under a laser confocal microscope for observation. The tobacco was acquired from the Tobacco Research Institute of Anhui Academy of Agricultural Sciences.

### Genetic Transformation of *GhArfGAP25* in *Arabidopsis thaliana*

*Arabidopsis thaliana* is a wild type of Columbia, which is planted on a pot containing nutrient soil and vermiculite (1:2) and cultured in an artificial climate room with a light:dark cycle of 16/8 h and a temperature of 22°C. Transgenic plants were generated by the *agrobacterium*-mediated transformation method (Zhang et al., 2006). The method used the recombinant plasmid mentioned above. Transgenic plants were selected on a plate culture medium containing 50 mg L^–1^ hygromycin. The hygromycin-resistant plants were transplanted and subsequently monitored for growth. Positive lines showed normal growth and true leaves, while the non-positive lines showed short plants, noeuphyllas, or even death.

### β-Glucuronidase Histochemical Analysis

Plant tissues were collected from *GhArfGAP25* transgenic *A. thaliana* lines. The tissues were placed in a 1.5-ml centrifuge tube, the pre-cooled 90% acetone was added to completely cover the material, and the leaves were treated at room temperature for 20 min. Then, the material was rinsed with distilled water, placed in a 1.5 ml-centrifuge tube, covered with the appropriate amount of β-Glucuronidase (GUS) staining solution (100 mmol L^–1^ NaH_2_ PO_4_ buffer pH 7.0, 0.5% Triton X-100, 0.5 mg mL^–1^X-Gluc, and 20% methanol), wrapped in tin foil papers, and placed overnight at 37°C. Tissues were subsequently rinsed by 95% ethanol, mounted on slides, and photographed using a stereomicroscope (Leica MZ95, Nussloch, Germany).

## Results

### Acquisition and Observation of the Boll Stalk Abscission Layer

**FIGURE 1:**
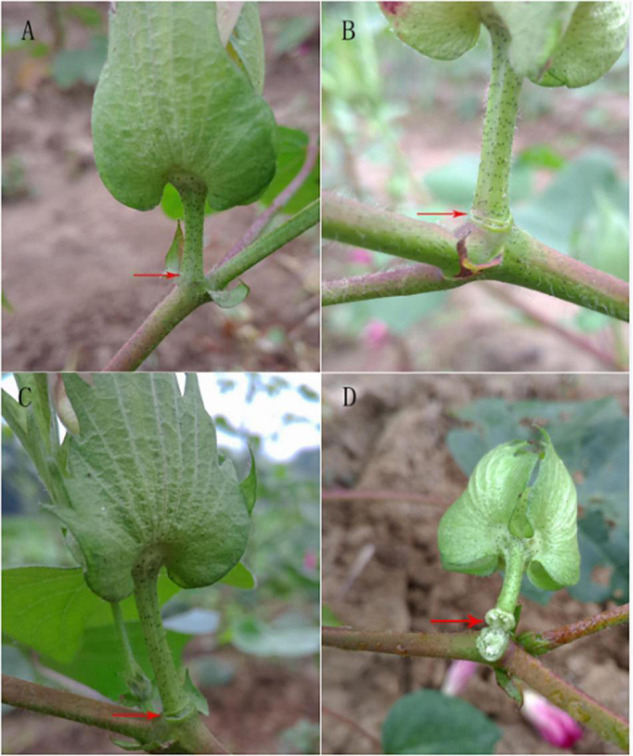
Effect of different concentrations of ethylene for 24 h on the abscission layer in the cotton pedicle. **(A)** The abscission layer of cotton treated with water for 24 h. **(B)** The abscission layer of cotton treated with 200 mg L^–1^ ethylene for 24 h. **(C)** The abscission layer of cotton treated with 400 mg L^–1^ ethylene for 24 h. **(D)** The abscission layer of cotton treated with 800 mg L^–1^ ethylene for 24 h. The red arrows refer to the abscission layer positions of the cotton pedicle.

Ethylene concentrations of 200, 400, and 800 mg L^–1^ were applied to cotton petioles with the same growth rate and at the full flowering stage. After 24 h, the effects of different concentrations of ethylene on the formation of the abscission layer of the flower stalks were observed (Figure 1). It can be seen that with the increase in ethylene concentration, the effect of the formation of the abscission layer was more obvious, but no abscission layer was found in water treatment. After being treated with 800 mg L^–1^ for 24 h, most of the petioles had fallen off. The effect of 200 mg L^–1^ treatment for 24 h on the formation of abscission was not significantly different from 400 mg L^–1^ treatment. Therefore, the cotton petiole treated with 400 mg L^–1^ ethylene was selected as the experimental material.

**FIGURE 2:**
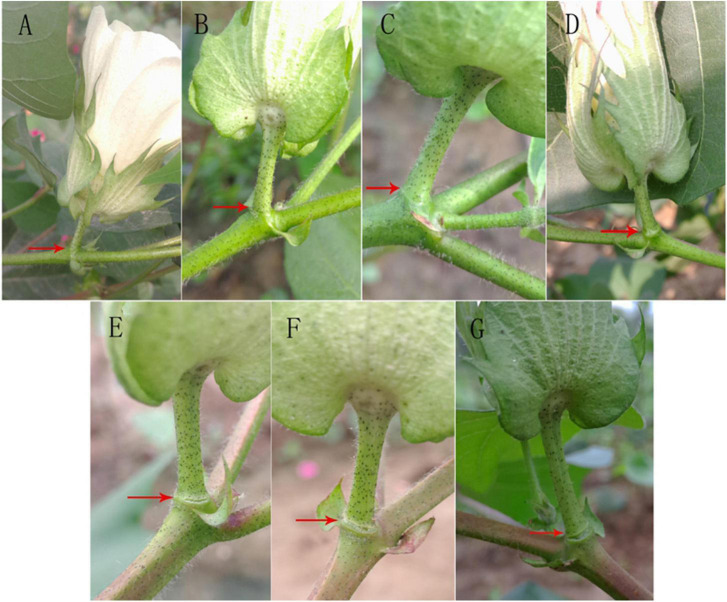
Effect of ethylene treatment at different times on the abscission layer in the cotton pedicle. **(A)** The abscission layer of cotton treated with 400 mg L^–1^ ethylene for 0 h. **(B)** The abscission layer of cotton treated with 400 mg L^–1^ ethylene for 4 h. **(C)** The abscission layer of cotton treated with 400 mg L^–1^ ethylene for 8 h. **(D)** Abscission layer of cotton treated with 400 mg L^–1^ ethylene for 12 h. **(E)** Abscission layer of cotton treated with 400 mg L^–1^ ethylene for 16 h. **(F)** Abscission layer of cotton treated with 400 mg L^–1^ ethylene for 20 h. **(G)** Abscission layer of cotton treated with 400 mg L^–1^ ethylene for 24 h. The red arrows refer to the abscission layer positions of the cotton pedicle.

After treatment with 400 mg L^–1^ ethylene, the change in the abscission layer at different time periods after treatment was observed (Figure 2). There was no significant change in the abscission layer of the boll stalk 8 h after treatment, but at 12 h, the change in the abscission layer is clear. There were broken marks between the boll stalk and the stem, and the abscission layer began to form. After 12 h, the broken marks gradually expanded, and finally, the petiole fell off.

**FIGURE 3:**
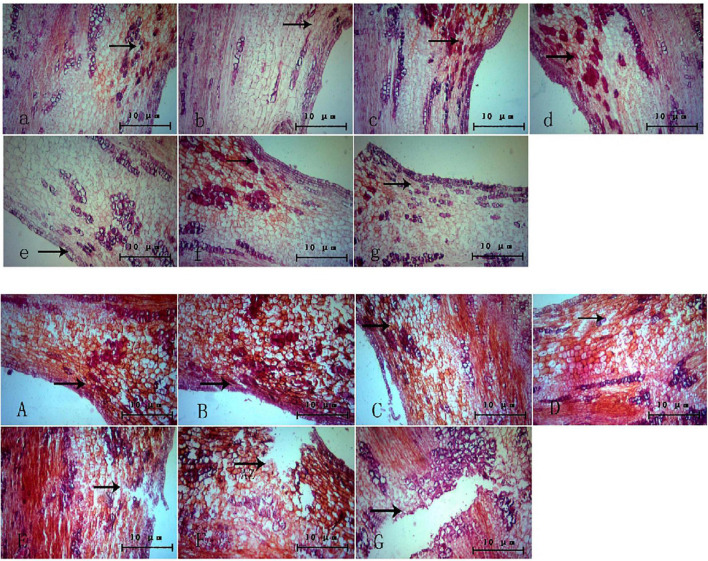
Microscopic observation of the abscission layer with different treatments at different times. Panels **(a–g)** were the control groups. Panels **(A–G)** were the experiment groups. Panels **(a–g)** show a microscopic image of the abscission layer treated with water at 0, 4, 8, 12, 16, 20, and 24 h, respectively. Panels **(A–G)** show a microscopic image of the abscission layer treated with ethylene 0, 4, 8, 12, 16, 20, and 24 h, respectively. The black arrow refers to the abscission layer position.

The changes in the abscission layer cells were observed using a microscope. In the control group (Figure 3), it can be observed that, with the passage of time, there is no obvious morphological difference between the cells in the abscission layer of the boll stalk and the surrounding cells, and the cells are closely arranged, indicating that water treatment has no effect on the abscission layer cells. However, in the treatment group (Figure 3), around 12–16 h after treatment, the cells became loose, the cell walls of the exfoliated cells began to disintegrate, and obvious tissue fracture marks appeared. As time went on, the fracture marks became larger and larger until the petiole exfoliated. It can also be seen from the figure that the formation of the abscission layer starts from the epidermal cells and extends to the vascular bundles of the boll stalk.

**FIGURE 4:**
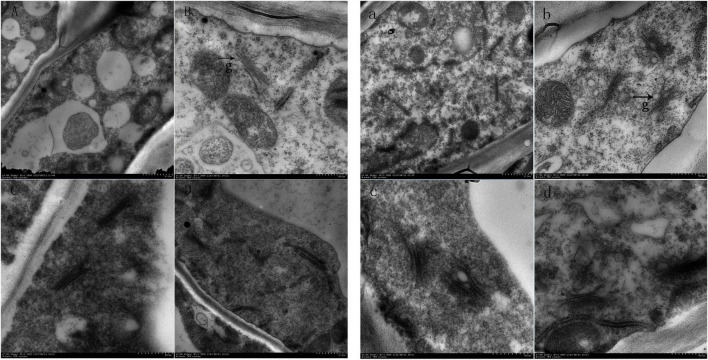
Electron microscopic observation on the abscission layer at different times. Panels **(A–D)** were the control groups. Panels **(a–d)** were the experiment groups. Panels **(A–D)** show electron microscopy images of the abscission layer treated with water 0, 8, 16, and 24 h, respectively. Panels **(a–d)** show electron microscopy images of the abscission layer treated ethylene 0, 8, 16, and 24 h, respectively. The black arrow with the letter g points to the Golgi position.

Scanning electron microscopy showed that the number of the Golgi apparatus and the number of stacking layers of the Golgi apparatus in the treatment group were significantly more than those in the control group (Figure 4). This indicated that the number and structure of the Golgi apparatus changed during the formation of the ionosphere that was induced by ethylene. The increased number of the Golgi apparatus and the stacking layers is related to many physiological and biochemical reactions in the process of the formation of the abscission layer.

### PhyloGenetic Tree Analysis, Chromosome Localization, and Protein Conserved Motif Analysis of *GhArfGAP* Gene Family

**FIGURE 5:**
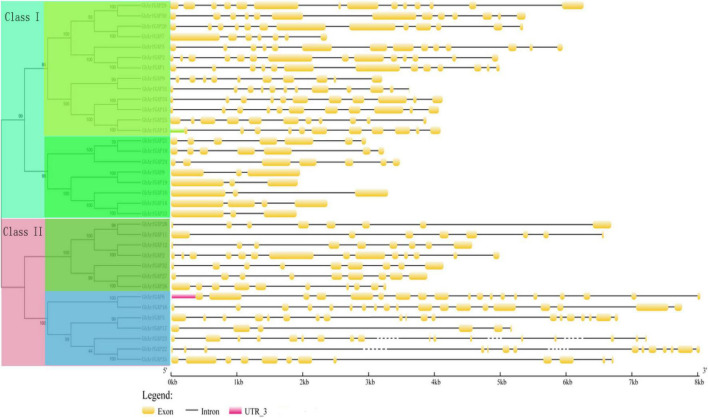
Phylogenetic tree and the structure of *GhArfGAP* genes in cotton. In the front half of this picture is a phylogenetic tree of the *GhArfGAP* genes. These genes are divided into two categories: Class I and Class II. The value represents the similarity. The back half of this picture is the analysis of the *GhArfGAP* genes’ structure. The yellow block indicates exon; the gray, solid, and dashed lines indicate introns; and the red block indicates 5’UTR. The ruler below indicates the length and direction of the gene. The unit is kilobase (Kb).

The characteristic domain of *the GhrfGAP* gene was obtained by the Pfam protein database. TblastN sequence comparison was carried out in the whole genome of cotton using “DNATOOLS” software. The *GhArfGAP* gene was compared by the cluster multi-sequence comparison tool in MEGA6.0 software, and repeated and redundant *GhArfGAP* genes were deleted, and then, the residual base was tested by Pfam and SMART. The amino acid sequence contains the GhArfGAP conserved domain. Finally, 35 candidate genes of *GhArfGAP* were obtained. According to their location on chromosomes, they are named GhGrfGAP1-GhGrfGAP35 (Figure 5). The molecular weight and isoelectric point of protein–amino acids encoded by the ExPasy proteomics server online tool were predicted in Supplementary Table 2. The results showed that the length of 35 GhArfGAP proteins was 275–867 amino acid residues, and the longest and the shortest was GhArfGAP27. The largest molecular weight is for GhArfGAP20, 94.1 KD. The smallest is for GhGrfGAP17, 30.9 KD. The maximum isoelectric point is GhArfGAP20, 9.38, and the minimum point is GhArfGAP11, 4.96.

**FIGURE 6:**
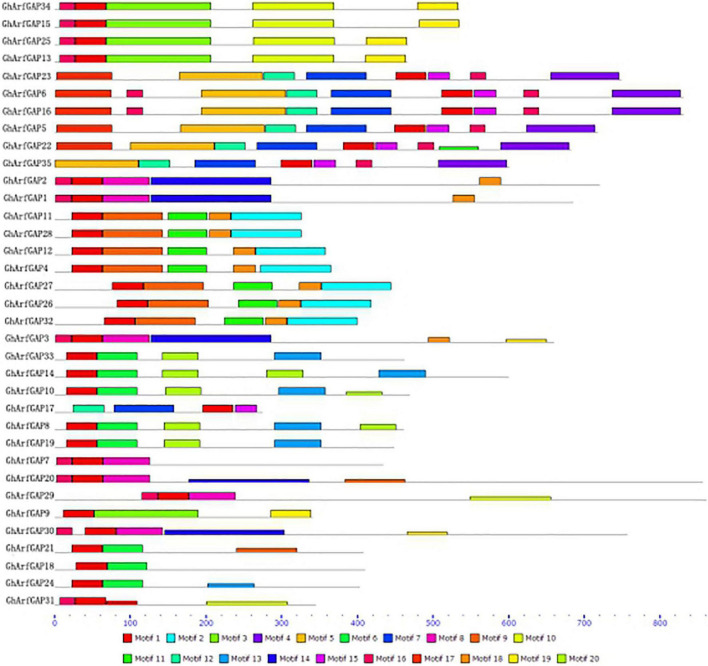
Motif distribution of GhArfGAP protein sequences in cotton. Rectangles of different colors represent different motifs. The gray line represents the amino acid sequence. The lower dashed ruler indicates the molecular weight of the protein. The unit is kilodalton (KD).

The MEME online analysis tool was used to analyze the conserved motifs of GhArfGAP protein in cotton (Figure 6). The results show that motif 1 exists in every GhArfGAP, which indicates that motif 1 may be necessary for GhArfGAP to play its function. Some motifs exist among some families, and others do not, such as motif 2 and motif 4, which indicate that these motifs are related to some functions of the family proteins. At the same time, we can find that the same family has the same motif type and order, but different families have differences. At present, the functions of motifs in the *GhArfGAP gene* family of cotton are not clear, but they may be necessary for these proteins to perform their functions.

**FIGURE 7:**
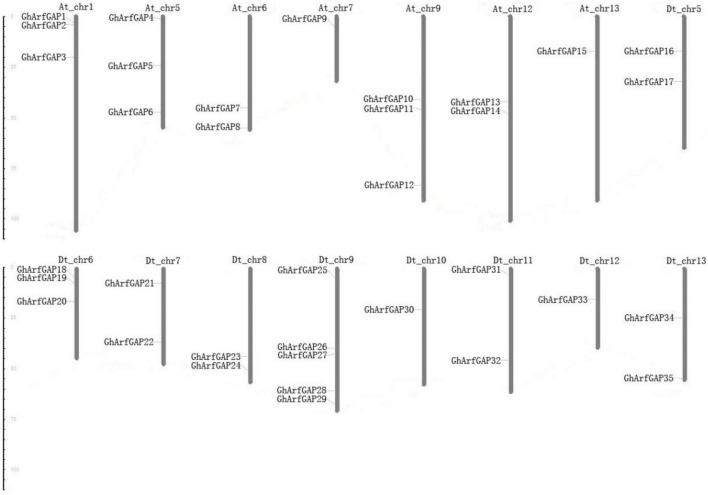
Chromosomal location of 35 *GhArfGAP* genes in cotton. At and Dt represent the A and D subgenomes of *Gossypium hirsutum*, respectively. Chr plus the number represents chromosome number. The ruler on the left represents the chromosome length. The unit is centimoles (cM).

The distribution of 35 *GhArfGAP* genes on chromosomes was generated by MapInspect software (Figure 7).

The results show that the 35 *GhArfGAP* genes were distributed on 16 of 26 chromosomes of cotton, and the gene distribution was relatively uniform in At and Dt genomes of cotton. Most of the *GhArfGAP* genes were distributed on chromosome 9 of DT genome, 5 *GhArfGAPs* were located on it, while only one GhArfGAP gene was found on chromosomes 7 and 13 and chromosomes 10 and 12 of At genome. It is believed that more than three genes are contained in the nucleotide units of about 200 kb, which is called gene cluster (Holub, 2001; Lv et al., 2017), but no gene cluster has been found on the chromosome of cotton.

### Relative Expression Analysis of *GhArfGAP13*, *GhArfGAP15*, *GhArfGAP25*, and *GhArfGAP34* in Different Tissues and Different Times After Ethylene Treatment

**FIGURE 8:**
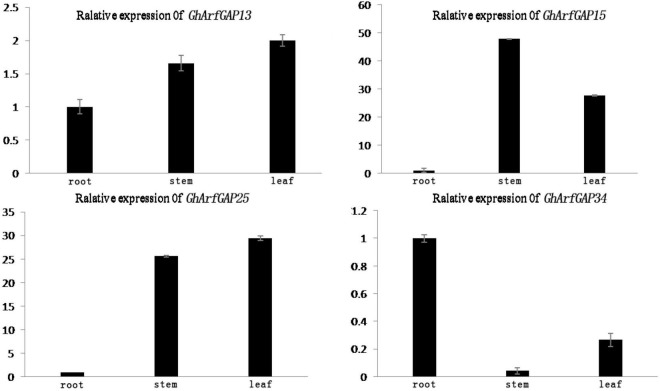
Tissue-specific expression analysis of *GhArfGAP13, GhArfGAP15, GhArfGAP25*, and *GhArfGAP34* in cotton. The expression of these four genes were detected in the roots, stems, and leaves. These tissue materials were taken from 400 mg⋅L^–1^ ethylene-treated cotton.

The phylogenetic tree analysis showed that *GhArfGAP13*, G*hArfGAP15*, G*hArfGAP25*, and G*hArfGAP34* were clustered into one group (Supplementary Figure 1) and suggested that these genes might be related to the formation of the abscission layer. Therefore, qRT-PCR was used to analyze the expression of these genes. The results (Figure 8) showed that *GhArfGAP13* was expressed in the roots, stems, and leaves, the highest expression level was found in leaves, about two times higher than that in roots, and the expression level in the stems was about 1.5 times that in the roots; *GhArfGAP15* expression was highest in the stems, but almost no expression was seen in the roots; *GhArfGAP25* expression was relatively high in the stems and leaves but almost no expression was seen in the roots; the expression level of *GhArfGAP34* was highest in the roots, relatively low in the leaves, and almost nil in the stems. These data indicated that *GhArfGAP13*, *GhArfGAP15*, *GhArfGAP25*, and *GhArfGAP34* were expressed in all tissues of cotton, but the expression levels in different tissues were different, which might be related to the functions of these genes in cotton.

**FIGURE 9:**
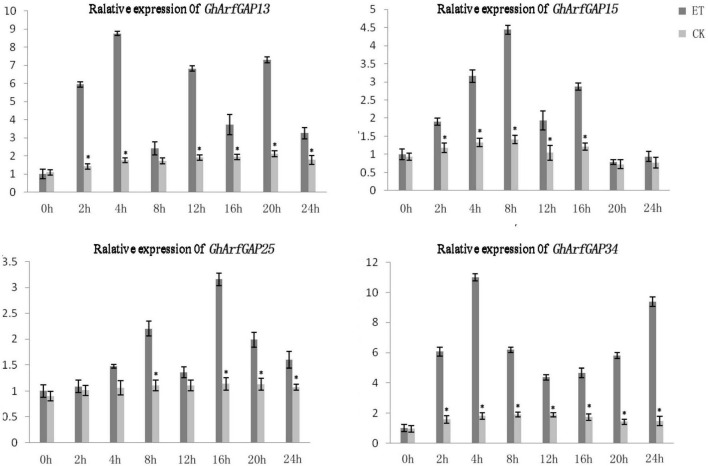
Different time expression analyses of *GhArfGAP13, GhArfGAP15*, *GhArfGAP25*, and *GhArfGAP34* in cotton. For *GhArfGAP13*, the asterisks indicate significant differences between ET and CK at 2, 4, 6, 12, 16, 20, and 24 h (*t*-test, *P* < 0.05). For *GhArfGAP15*, the asterisks indicate significant differences between ET and CK at 2, 4, 6, 8, 12, and 16 h (*t*-test, *P* < 0.05). For *GhArfGAP25*, the asterisks indicate significant differences between ET and CK at 8, 16, 20, and 24 h (*t*-test, *P* < 0.05). For *GhArfGAP34*, the asterisks indicate significant differences between ET and CK at 2, 4, 6, 8, 12, 16, 20, and 24 h (*t*-test, *P* < 0.05). The experimental materials were leaves taken from 400 mg⋅L^–1^ ethylene-treated cotton.

Furthermore, the expression level of these four genes in different time periods of the formation of ethylene-induced abscission was detected (Figure 9). In general, the expression levels of *GhArfGAP15* and *GhArfGAP25* genes increased first and then decreased, and both showed a downward trend at 12 h. The difference was that the peak of *GhArfGAP15* expression appeared at 8 h, and the peak of *GhArfGAP25* expression appeared at 16 h; the expression levels of *GhArfGAP13* showed an alternating rise and fall, and the peak appeared at 4 h. The expression level of *GhArfGAP34* increased first, then decreased, and then increased, and the peak appeared at 4 h, but the expression level of *GhArfGAP13* at 0 h was lower than that of the control group, and the other expression levels were higher than those of the corresponding control group. There was no significant change in the expression of the four genes in the control group. The changed expression of *GhArfGAP13*, *GhArfGAP15*, *GhArfGAP25*, and *GhArfGAP34* indicated that these four genes all played a certain role in the formation of the abscission layer.

### Structural Analysis of GhArfGAP25 Protein

According to the results of qRT-PCR, we cloned *GhArfGAP25* and expected to study its role in the formation of the abscission layer. The structure of the protein expressed by this gene was analyzed. TMHMM tool analysis showed that the protein had no transmembrane domain (Supplementary Figure 1A). An online tool, signalP 4.1 server, predicted that the GhArfGAP25 protein does not contain a signal peptide sequence, and it is a non-secretory protein (Supplementary Figure 1B). Studies have shown that ArfGAP protein is involved in intracellular material transport, which indirectly proves that the prediction is reliable (Liu et al., 2001; Dan and Kahn, 2004). The analysis of the secondary structure of the GhArfGAP25 protein by NPS showed (Supplementary Figure 1C) that the GhArfGAP25 protein is mainly composed of α-helix and random coil, in addition to some β-turns and extended chain. There are 158 α-helices, accounting for 33.83% of the total protein; 237 irregular curls, accounting for 50.75% of the total protein; 25 β-turns, accounting for 5.35% of the total protein; and 47 extended chains, accounting for 10.06% of the total protein. SWISS-MODEL (Konstantin et al., 2006; Florian et al., 2008; Guex et al., 2010; Marco et al., 2014) was used to predict the tertiary structure of the GhArfGAP25 protein. The results are shown in Supplementary Figure 1D. The main structure was α-helix, irregular curl, and a small number of β-turns.

### Analysis of Expression in Transgenic *Arabidopsis thaliana* Plants

**FIGURE 10:**
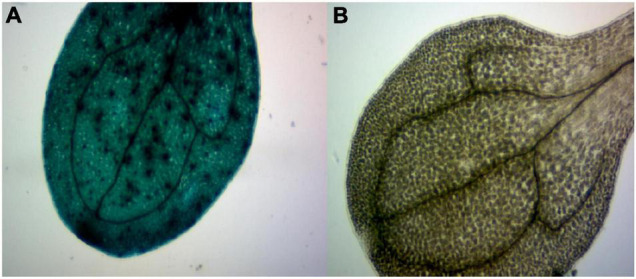
β-Glucuronidase (GUS) staining of transgenic plants. **(A)** The image of a stained transgenic *Arabidopsis thaliana* leaf. **(B)** The image of a stained wild-type *A. thaliana* leaf.

In order to further study *GhArfGAP25*, we transferred *GhArfGAP25* into *A. thaliana* by *Agrobacterium* transformation, which was verified by GUS staining (Figure 10). Fortunately, we got transgenic *A. thaliana*.

**FIGURE 11:**
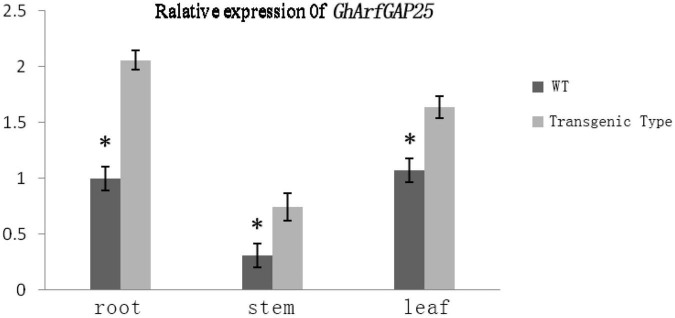
Expression analysis of *GhArfGAP25* in different tissues of transgenic *Arabidopsis thaliana* plants. The asterisks indicate significant differences between transgenic *A. thaliana* and WT in the roots, stems, and leaves (*t*-test, *P* < 0.05).

We analyzed the expression of *the GhArfGAP25 gene* in different tissues of wild-type and transgenic *A. thaliana* by qRT-PCR (Figure 11). The results showed that the expression of *GhArfGAP25* in the roots, stems, and leaves of transgenic *A. thaliana* was higher than that of wild type. Compared with different tissue parts of cotton, the expression of *the GhArfGAP25 gene* was the lowest in the *A. thaliana* stem and the highest in the root, while the expression of *the GhArfGAP25 gene* was the lowest in cotton root and the highest in the leaf, indicating that the expression of the same gene was different in different tissue parts of different species.

### The Function of *GhArfGAP25* Is Located in the Golgi Apparatus and Endoplasmic Reticulum

**FIGURE 12:**
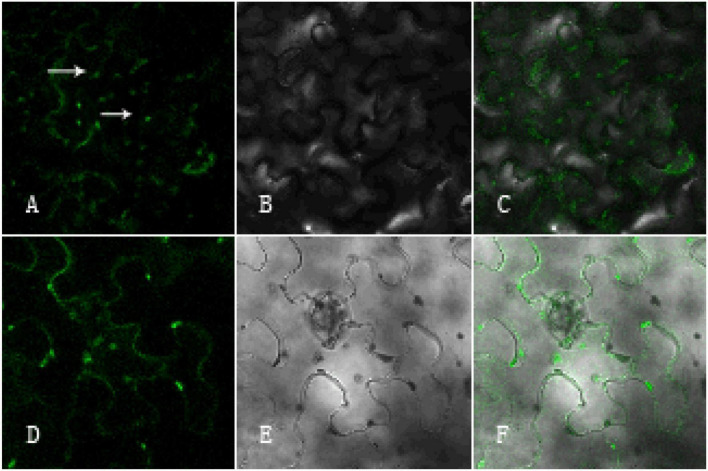
Subcellular localization of pCambia1304-*GhArfGAP25* and pCambia1304. **(A–C)** Subcellular localization of pCambia1304-*GhArfGAP25*; **(D–F)** subcellular localization of pCambia1304 as a control group; **(A,D)** fluorograph; **(B,E)** micrograph; **(C,F)** merge.

To investigate the functional localization of the *GhArfGAP25* gene, tobacco was used for subcellular localization (Figure 12). The results showed that the green fluorescence was the fluorescence emitted by GFP and the surrounding tobacco epidermal cells. The green fluorescence emitted by GhArfGAP25-GFP not only appeared around the tobacco leaf epidermal cell membrane but also appeared in the cells. Therefore, it was speculated that the function of *the GhArfGAP25* gene was located in the Golgi apparatus and endoplasmic reticulum, which was consistent with the previous studies.

## Discussion

### Formation of the Abscission Layer of the Cotton Boll Stalk Induced by Ethylene

Ethylene is an essential hormone in plants, which plays a momentous role in the whole process of plant growth and development (Tsai et al., 2016). At the same time, ethylene can also induce the formation of the abscission layer and can cause the abscission of flowers and fruits. In mango, after ethylene induction, the expression of *MiIDA1* and *MiIDA2* had upregulated and the formation of the abscission layer was promoted (Rai et al., 2021). In *Camellia oleifera, CoIDAs* are abscission-associated genes, which can be induced by ethylene and push forward the formation of the abscission layer (Yang et al., 2021). Our previous studies have shown that ethylene treatment stimulates the formation of the abscission layer in cotton, and the genome-wide analysis showed that it was related to miRNA (Guo et al., 2018). In this experiment, after 12 h of treatment with 400 mg⋅L^–1^ ethylene, the morphology of the abscission layer cells changed, the arrangement of cells became loose, the cell wall began to degrade and separate from the surrounding cells, and the abscission layer began to form. During the formation of the abscission layer, a series of physiological and biochemical reactions occur in cells, and a large number of molecules need to be synthesized and transported. Hence, the number of the Golgi apparatus and the number of the abscission layer increased. Studies have shown that the main function of the Golgi apparatus is to process, classify, and package proteins synthesized by the endoplasmic reticulum and then send them to specific parts of cells or secrete them out of cells (Pfeffer and Rothman, 1987).

### *GhArfGAP* Gene Function May Be Related to the Golgi Apparatus and Vesicle Trafficking

A number of reports support the hypothesis that ArfGAPs are involved in vesicle trafficking at the Golgi apparatus. In yeast, ArfGAP Age1 is regulated by phospholipase D for post-Golgi vesicular transport (Benjamin et al., 2011). In rice, overexpression of *OsAGAP* caused pattern changes in vesicle trafficking (Zhuang et al., 2010a). *OsAGAP* can also recover the defect of vesicular transport in the yeast ArfGAP double mutant *gcs1Dglo3D* (Zhuang et al., 2010b). In the qRT-PCR analysis of the expression of *GhArfGAP13*, *GhArfGAP15*, *GhArfGAP25*, *and GhArfGAP34* levels, in the cotton root, *GhArfGAP13* and *GhArfGAP34* expression levels were higher and *GhArfGAP15* and *GhArfGAP25* expression levels were lower. At the same time, the expression level of the *GhArfGAP25* gene in transgenic *A. thaliana* was the highest in roots. After ethylene induction, the number of the isolated Golgi apparatus and the stacking layers increased significantly, and the expression levels of these four genes were higher than those of the control group. Based on these results and the related literature, we speculated that *GhArfGAP*, in particular *GhArfGAP25*, is involved in the Golgi apparatus vesicular transport and root growth and development in cotton, but further research is needed.

## Conclusion

In this study, ethylene could induce the formation of the abscission layer in *Gossypium hirsutum* and then promote boll stalk detaching, and the number and the structure of the Golgi apparatus in the abscission layer also changed. *GhArfGAP13, GhArfGAP15, GhArfGAP25*, and *GhArfGAP34* might regulate the changes in the Golgi apparatus. Our study provides new insight into the regulatory mechanisms for the formation of the abscission layer and for improving cotton production.

## Data Availability Statement

The original contributions presented in this study are included in the article/Supplementary Material, further inquiries can be directed to the corresponding author/s.

## Author Contributions

NG and SY conceived and designed the experiments. AL, LC, and ZG performed the experiments. HJ, LL, DL, and JG analyzed the data. LC wrote the manuscript. All authors have read and approved the manuscript.

## Conflict of Interest

The authors declare that the research was conducted in the absence of any commercial or financial relationships that could be construed as a potential conflict of interest.

## Publisher’s Note

All claims expressed in this article are solely those of the authors and do not necessarily represent those of their affiliated organizations, or those of the publisher, the editors and the reviewers. Any product that may be evaluated in this article, or claim that may be made by its manufacturer, is not guaranteed or endorsed by the publisher.

## Abbreviations

ArfGAP: ADP ribosylation factor GTPase-activating protein
UTR: untranslated region
miRNA: microRNA
GFP: green fluorescent protein.
