# Supplementary material for: Cloning and Bioinformatics Analysis of GhArfGAP in Cotton (Gossypium hirsutum) Boll Abscission Layer With Ethylene Treatment
Source: Front Plant Sci. 2022 Jun 24;13:841161. doi: 10.3389/fpls.2022.841161 (PMC9263981; doi:10.3389/fpls.2022.841161)

| Primer name | Primer sequence |
| --- | --- |
| *GhArf*GAP13-F | 5’-CGATTATGCTACAGACCTT-3’ |
| *GhArfGAP13-R* | 5’-ATCATCAGTAGAAGTTGCC-3’ |
| *GhArf GAP15-F* | 5’-TGAGTATGCTATGGACCT-3’ |
| *GhArfGAP15-R* | 5’-CAATCATTATCATCAGTGGAA-3’ |
| *GhArf GAP25-F* | 5’-CTGCTACACTAGACACAT-3’ |
| *GhArfGAP25-R* | 5’-CGGATGAAGTTCTCAATTC-3’ |
| *GhArf GAP34-F* | 5’-TGCAAATAGGACAGACTCAACA-3’ |
| *GhArfGAP34-R* | 5’-GCAACACTGCTTGCTGGG-3’ |
| *UBQ7-F* | 5’-TCGAACCAACCGACACTA-3’ |
| UBQ7-R | 5’-GCCTCCCTCTATCGCATA-3’ |

TableS1 Table S1. Primer list

TableS2

Table S2. Identification and related information of *GhArfGAP gene* family in upland cotton

| Gene | Protein ID | Protein length | Molecular weight/KD | Theoretical pI | Chromosome |
| --- | --- | --- | --- | --- | --- |
| GhArfGAP1 | CotAD_34498 | 685 | 75106.9 | 5.08 | At_chr1 |
| GhArfGAP2 | CotAD_35891 | 720 | 78931.3 | 5.13 | At_chr1 |
| GhArfGAP3 | CotAD_72535 | 660 | 72391.9 | 6.32 | At_chr1 |
| GhArfGAP4 | CotAD_28193 | 365 | 40516.4 | 4.99 | At_chr5 |
| GhArfGAP5 | CotAD_53305 | 718 | 80702.6 | 7.51 | At_chr5 |
| GhArfGAP6 | CotAD_65604 | 831 | 92818.5 | 7.12 | At_chr5 |
| GhArfGAP7 | CotAD_42298 | 434 | 49824.7 | 9.23 | At_chr6 |
| GhArfGAP8 | CotAD_18859 | 461 | 50995.2 | 8.86 | At_chr6 |
| GhArfGAP9 | CotAD_41245 | 341 | 37418 | 6.06 | At_chr7 |
| GhArfGAP10 | CotAD_03468 | 469 | 51107.8 | 8.87 | At_chr9 |
| GhArfGAP11 | CotAD_29975 | 326 | 35809.6 | 4.96 | At_chr9 |
| GhArfGAP12 | CotAD_05792 | 358 | 39797.6 | 4.99 | At_chr9 |
| GhArfGAP13 | CotAD_62373 | 466 | 50468.5 | 6.68 | At_chr12 |
| GhArfGAP14 | CotAD_73701 | 600 | 64800 | 6.03 | At_chr12 |
| GhArfGAP15 | CotAD_28095 | 537 | 57899.8 | 8.21 | At_chr13 |
| GhArfGAP16 | CotAD_02215 | 831 | 92834.4 | 6.83 | Dt_chr5 |
| GhArfGAP17 | CotAD_24647 | 275 | 30960 | 8.29 | Dt_chr5 |
| GhArfGAP18 | CotAD_11419 | 410 | 44363.8 | 8.79 | Dt_chr6 |
| GhArfGAP19 | CotAD_03357 | 449 | 49929.1 | 9.08 | Dt_chr6 |
| GhArfGAP20 | CotAD_22552 | 857 | 94099.7 | 9.38 | Dt_chr6 |
| GhArfGAP21 | CotAD_03958 | 408 | 43923 | 8.9 | Dt_chr7 |
| GhArfGAP22 | CotAD_24203 | 683 | 76152.5 | 5.84 | Dt_chr7 |
| GhArfGAP23 | CotAD_13101 | 749 | 84067 | 5.98 | Dt_chr8 |
| GhArfGAP24 | CotAD_66253 | 403 | 43753 | 9.02 | Dt_chr8 |
| GhArfGAP25 | CotAD_24323 | 467 | 50631.7 | 6.48 | Dt_chr9 |
| GhArfGAP26 | CotAD_02512 | 418 | 47385.1 | 7.99 | Dt_chr9 |
| GhArfGAP27 | CotAD_63912 | 445 | 50610.6 | 6.51 | Dt_chr9 |
| GhArfGAP28 | CotAD_01587 | 326 | 35778.6 | 5.1 | Dt_chr9 |
| GhArfGAP29 | CotAD_00490 | 867 | 94098.2 | 8.45 | Dt_chr9 |
| GhArfGAP30 | CotAD_23832 | 757 | 81715.3 | 6.7 | Dt_chr10 |
| GhArfGAP31 | CotAD_22189 | 345 | 38322.1 | 8.71 | Dt_chr11 |
| GhArfGAP32 | CotAD_46685 | 400 | 44849.7 | 7.62 | Dt_chr11 |
| GhArfGAP33 | CotAD_19029 | 462 | 50097.3 | 6.06 | Dt_chr12 |
| GhArfGAP34 | CotAD_06070 | 535 | 57737.6 | 7.7 | Dt_chr13 |
| GhArfGAP35 | CotAD_30732 | 601 | 67051.2 | 6.22 | Dt_chr13 |

，

Fig.S1


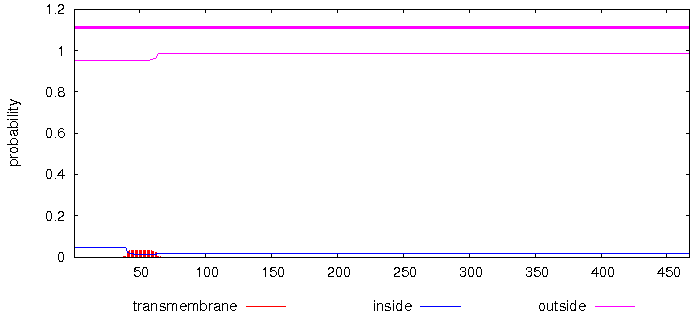

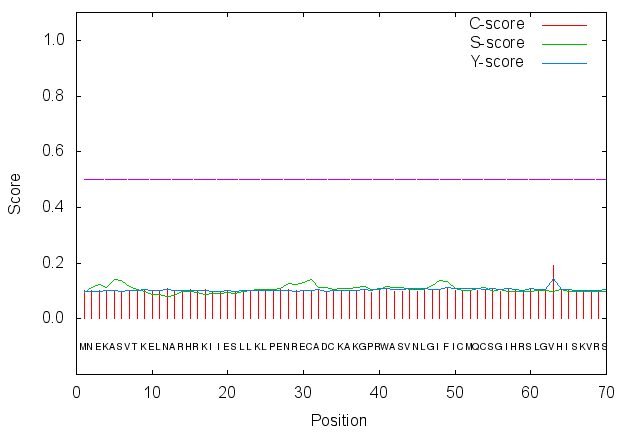


A

C

B


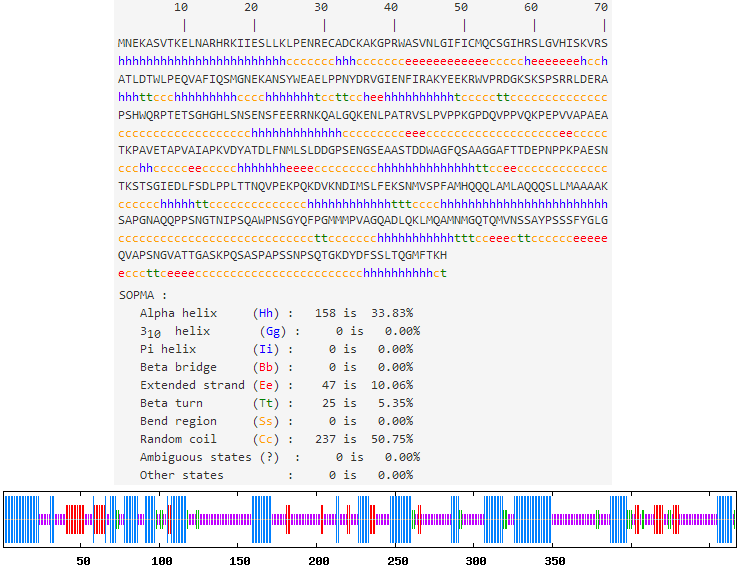

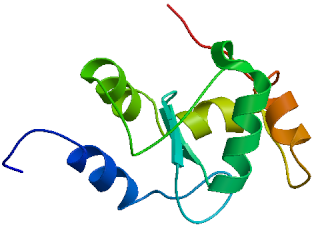


D

Fig.S1 Structural analysis o fGhArfGAP25 protein.( A)transmembrane domain prediction of GhArfGAP25 protein.

(B)predicted signal peptide of GhArfGAP25 (C score: The score for origihal cleavage site;Y score: The score for general cleavage site;S score: The score for signal peptide).(C)secondary structure prediction of GhArfGAP25 protein (Hh: Alpha helix; Ee: Extended strand; Tt: Beta turn; Cc: Random coil).(D)predicted tertiary structure of the GhArfGAP25 protein.


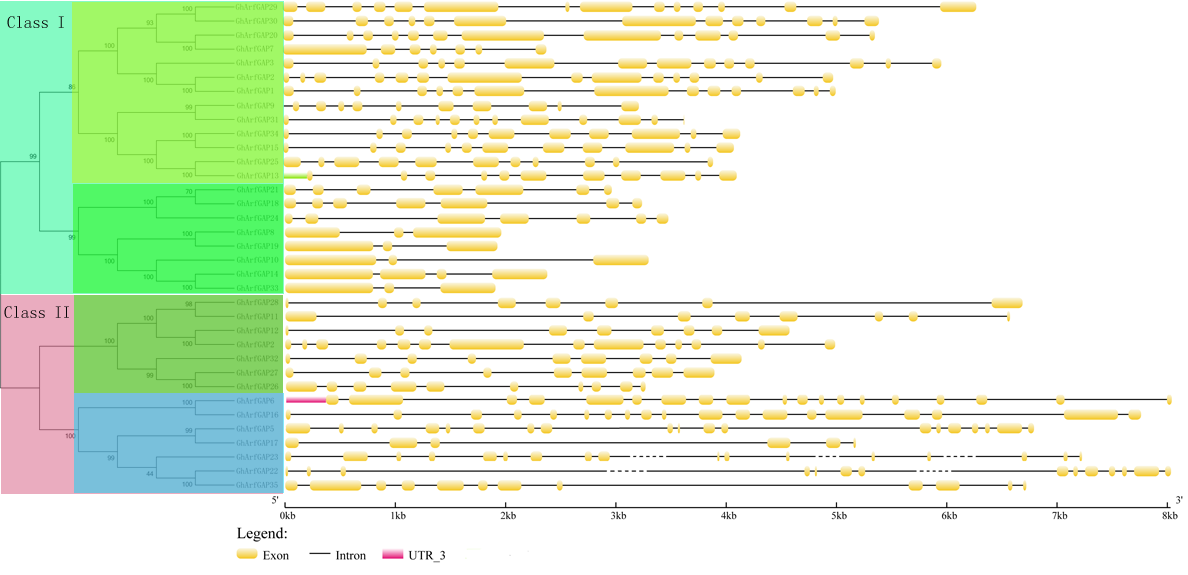

Supplement: Supplementary file 1 [file Data_Sheet_1.doc]
